# Supplementary material for: MouseGoggles: an immersive virtual reality headset for mouse neuroscience and behavior
Source: Nat Methods. 2024 Dec 12;22(2):380–5. doi: 10.1038/s41592-024-02540-y (PMC11810773; doi:10.1038/s41592-024-02540-y)
Supplement: Supplementary file 1 — Reporting Summary [file 41592_2024_2540_MOESM1_ESM.pdf]

nature portfolio

Corresponding author(s): Matthew Isaacson

Last updated by author(s): Oct 24, 2024

Reporting Summary

Nature Portfolio wishes to improve the reproducibility of the work that we publish. This form provides structure for consistency and transparency in reporting. For further information on Nature Portfolio policies, see our Editorial Policies and the Editorial Policy Checklist.

Statistics

For all statistical analyses, confirm that the following items are present in the figure legend, table legend, main text, or Methods section.

n/a	Confirmed	
☐	☒	The exact sample size (*n*) for each experimental group/condition, given as a discrete number and unit of measurement
☐	☒	A statement on whether measurements were taken from distinct samples or whether the same sample was measured repeatedly
☐	☒	The statistical test(s) used AND whether they are one- or two-sided<br>*Only common tests should be described solely by name; describe more complex techniques in the Methods section.*
☒	☐	A description of all covariates tested
☐	☒	A description of any assumptions or corrections, such as tests of normality and adjustment for multiple comparisons
☐	☒	A full description of the statistical parameters including central tendency (e.g. means) or other basic estimates (e.g. regression coefficient) AND variation (e.g. standard deviation) or associated estimates of uncertainty (e.g. confidence intervals)
☐	☒	For null hypothesis testing, the test statistic (e.g. *F*, *t*, *r*) with confidence intervals, effect sizes, degrees of freedom and *P* value noted<br>*Give P values as exact values whenever suitable.*
☒	☐	For Bayesian analysis, information on the choice of priors and Markov chain Monte Carlo settings
☒	☐	For hierarchical and complex designs, identification of the appropriate level for tests and full reporting of outcomes
☒	☐	Estimates of effect sizes (e.g. Cohen's *d*, Pearson's *r*), indicating how they were calculated

Our web collection on statistics for biologists contains articles on many of the points above.

Software and code

Policy information about availability of computer code

Data collection	All custom code used for data collection is available upon request, with the following open and commercial software dependencies listed here:<br>Raspberry Pi OS 32-bit (VR display operating system)<br>Godot 3.2.3.stable.flathub (VR experiment creation and rendering)<br>Arduino 1.8.15 (monocular display control; VR display I/O communication)<br>Teensyduino 1.57 (monocular display control; VR display I/O communication)<br>Matlab 2022b (imaging acquisition and visual stimulus control)<br>ScanImage SI2022 (Imaging acquisition)<br>https://github.com/adafruit/Adafruit-GFX-Library (visual stimulus generation)<br>https://github.com/juj/fbcp-ili9341 (SPI display driver)<br>https://github.com/Lauszus/ADNS308 (spherical treadmill motion tracking)<br>https://github.com/dmadison/ArduinoXInput (VR display I/O communication)
Data analysis	All custom code used for data analysis is available upon request, with the following open and commercial software dependencies listed here:<br>Matlab 2022b (general data analysis)<br>Python 2.8.8 (general data analysis)<br>suite2p v0.10.3 (suite2p.org) (calcium imaging analysis)<br>https://github.com/lolaBerkowitz/SNLab_ephys (electrophysiology analysis)<br>https://github.com/nelpy/nelpy (electrophysiology analysis)<br>https://github.com/ryanharvey1/neuro_py (electrophysiology analysis)

https://github.com/dnafinder/cuzick (Cuzick's statistical test for trend)
https://github.com/cortex-lab/KiloSort (electrophysiological spike sorting)
https://github.com/kwikteam/phy (curation of electrophysiology data)
https://github.com/petersenpeter/phy-plugins (curation of electrophysiology data)

For manuscripts utilizing custom algorithms or software that are central to the research but not yet described in published literature, software must be made available to editors and reviewers. We strongly encourage code deposition in a community repository (e.g. GitHub). See the Nature Portfolio guidelines for submitting code & software for further information.

Data

Policy information about availability of data

All manuscripts must include a data availability statement. This statement should provide the following information, where applicable:

- Accession codes, unique identifiers, or web links for publicly available datasets
- A description of any restrictions on data availability
- For clinical datasets or third party data, please ensure that the statement adheres to our policy

All data used in this manuscript is available upon request. Large datasets are deposited in the Figshare database at https://doi.org/10.6084/m9.figshare.24039021.v4.

Human research participants

Policy information about studies involving human research participants and Sex and Gender in Research.

Reporting on sex and gender	NA
Population characteristics	NA
Recruitment	NA
Ethics oversight	NA

Note that full information on the approval of the study protocol must also be provided in the manuscript.

Field-specific reporting

Please select the one below that is the best fit for your research. If you are not sure, read the appropriate sections before making your selection.

☒ Life sciences ☐ Behavioural & social sciences ☐ Ecological, evolutionary & environmental sciences

For a reference copy of the document with all sections, see nature.com/documents/nr-reporting-summary-flat.pdf

Life sciences study design

All studies must disclose on these points even when the disclosure is negative.

Sample size	No sample size calculation was performed. Sample sizes were based on previous scientific literature in the respective fields of study (Niell et al, J Neurosci 2008; Tan et al, Sci Rep 2015; Busse et al, J Neurosci 2011; Dombeck et al, Nat Neurosci 2010) using a minimum number of mice to successfully replicate previous findings.
Data exclusions	For linear track place learning experiments where 40 track traversal took place per session, some session exceeded 40 laps due to program glitches -- these extra trials were discarded. For all other experiments, no data was excluded from analysis.
Replication	For electrophysiology experiments, replication was performed with 2 mice. For all other experiments, replication was performed with at least 3 mice. All replication attempts were successful. For the looming visual stimulus experiment where a novel startle response was observed with an initial dataset of 2 mice, additional replication was attempted (and successful) with an additional 6 mice.
Randomization	For the looming visual stimulus experiment, mice were randomly allocated to either headset-first or projector-first experimental conditions. For linear track place learning experiment, mice were randomly allocated to the reward zone A vs reward zone B conditions. For all other experiments, randomization is not relevant for our study as they did not have multiple experimental groups.
Blinding	For mouse behavioral scoring of looming visual stimulus experiment, 2 independent scorers were blinded where possible; scorers were blinded to the purpose and details of the experiment, though they could not be completely blinded to the experimental condition (headset vs projector) due to the nature of the recorded videos which include equipment specific to each condition. All other data collection was performed through objective and unbiased automated data collection pipelines where blinding is not relevant.

Reporting for specific materials, systems and methods

We require information from authors about some types of materials, experimental systems and methods used in many studies. Here, indicate whether each material, system or method listed is relevant to your study. If you are not sure if a list item applies to your research, read the appropriate section before selecting a response.

Materials & experimental systems

n/a	Involved in the study
☒	☐ Antibodies
☒	☐ Eukaryotic cell lines
☒	☐ Palaeontology and archaeology
☐	☒ Animals and other organisms
☒	☐ Clinical data
☒	☐ Dual use research of concern

Methods

n/a	Involved in the study
☒	☐ ChIP-seq
☒	☐ Flow cytometry
☒	☐ MRI-based neuroimaging

Animals and other research organisms

Policy information about studies involving animals; ARRIVE guidelines recommended for reporting animal research, and Sex and Gender in Research

Laboratory animals	Animals used:<br>C57BL/6 (3 females, 35 males; 2-16 months old)<br>APPnl-g-f heterozygotes (3 males, 4 females; 2-3 months old)<br>TH::Cre heterozygotes (line FI12, www.gensat.org) (1 male; 16 months old)<br>Drd2::Cre heterozygotes (line ER44, www.gensat.org) (1 male, 2 females; 4 months old)<br>All animal procedures complied with relevant ethical regulations and were performed after approval by the Institutional Animal Care and Use Committee (IACUC) of Cornell University (protocol number 2015-0029). All mice were housed in a climate-controlled facility kept at 22o C and 40-50% humidity, under a 12-hour light-dark cycle with ad libitum access to food and water.
Wild animals	Study did not involve wild animals
Reporting on sex	For mouse inter-eye distance measurement, as this was a novel dataset, both male and female mice were used and data is reported disaggregated for sex. For all other experiments, only male mice were used to reduce variance in measurement and because these experiments replicated or extended results from prior publications where data on male mice were reported.
Field-collected samples	Study did not involve samples collected from the field
Ethics oversight	Ethics oversight provided by the Institutional Animal Care and Use Committee (IACUC) of Cornell University (protocol number 2015-0029).

Note that full information on the approval of the study protocol must also be provided in the manuscript.
